# A Semantic Approach with Decision Support for Safety Service in Smart Home Management

**DOI:** 10.3390/s16081224

**Published:** 2016-08-03

**Authors:** Xiaoci Huang, Jianjun Yi, Xiaomin Zhu, Shaoli Chen

**Affiliations:** 1 Shanghai University of Engineering Science, Shanghai 201620, China; 2 Department of Mechanical Engineering, East China University of Science and Technology, Shanghai 200237, China; zxmin4236@163.com (X.Z.); pariscat@aliyun.com (S.C.)

**Keywords:** smart homes, decision, safety service

## Abstract

Research on smart homes (SHs) has increased significantly in recent years because of the convenience provided by having an assisted living environment. The functions of SHs as mentioned in previous studies, particularly safety services, are seldom discussed or mentioned. Thus, this study proposes a semantic approach with decision support for safety service in SH management. The focus of this contribution is to explore a context awareness and reasoning approach for risk recognition in SH that enables the proper decision support for flexible safety service provision. The framework of SH based on a wireless sensor network is described from the perspective of neighbourhood management. This approach is based on the integration of semantic knowledge in which a reasoner can make decisions about risk recognition and safety service. We present a management ontology for a SH and relevant monitoring contextual information, which considers its suitability in a pervasive computing environment and is service-oriented. We also propose a rule-based reasoning method to provide decision support through reasoning techniques and context-awareness. A system prototype is developed to evaluate the feasibility, time response and extendibility of the approach. The evaluation of our approach shows that it is more effective in daily risk event recognition. The decisions for service provision are shown to be accurate.

## 1. Introduction

Recently, an increasing number of smart equipment has been used in homes to provide assistance with daily living activities. These smart appliances can “sense” the home environment, and consequently, improve convenience, comfort and safety. A smart home (SH) links these pieces of equipment (such as audio equipment, video equipment, light equipment, air-condition control and window curtain control) through the Internet of Things to provide equipment automation, time control and information interaction [1]. By monitoring environmental changes and the activities of inhabitants, an assistive system in an SH can process sensor data, infer the needs of an inhabitant and take appropriate actions to help the inhabitant perform daily living activities [2]. For this purpose, taking advantage of the latest SH technologies, studies about context-aware reasoning such as recognition of Activities of Daily Living (ADL) or service provision for some specific purposes (such as disable help) are being developed to enhance their quality of life. The employed technologies and targeted situations in the literature are diverse [3,4], but they all attempt to increase the end-user’s quality of life by providing smarter service for residents in SH through knowledge reasoning, technology-based solutions.

In this line, the usage of Web Ontology Language (OWL), in a particular semantic context expression, given the basis of satisfying context-aware reasoning, has been studied to achieve these target situations [5]. The study had shown that when ontological techniques were extended with temporal information, their effectiveness is higher compared with other methods [4]. Nevertheless, it seems there is still a long way to go to provide services which fully satisfy their safety assistance function. Therefore, for the purpose of providing service for residents to enhance the quality of life within their own homes, such as safety services, the SH needs to have enhanced reasoning capabilities based on environmental monitoring, equipment sensing and control, human activity recognition, and so on. The level of risk within a situation should be accurately identified in order to perform tasks more effectively. Safety assistance services should be performed and especially obey complex rules that may change with time and even depend on a specific situation. Therefore, a flexible solution that enables SHs to process a risk situation and provide decision support for safety assistance service incorporating complex and dynamic rules is particularly necessary.

The focus of this contribution is on exploring a context awareness and reasoning approach for risk event recognition in SH that enables the proper decision support for flexible safety assistance service. A semantic reasoning approach is proposed for risk recognition based on the knowledge description in OWL and rules designed with Semantic Web Rule Language (SWRL). In addition, the ontology is built from the perspective of flexible service. Combined with relevant reasoning rules, it is possible to infer a preferred service depending on the type of risk event. The evaluation of our approach shows that it has a good accuracy rate (75.95%) in daily risk recognition, and the decisions for service provision are found to be accurate. The approach typically implements effective strategies to help in the management of a neighbourhood which reduces the chance of accidents occurring within a home unit.

The remainder of this paper is organised as follows. The next section provides an overview of related SHs. In Section 3, the framework and methodology of the realization of SH management system from a safety perspective are discussed. In Section 4, we present our reasoning method for risk recognition and service decision support. Section 5 describes the workflow of the proposed approach in the system and an evaluation of the approach is conducted in Section 6. Section 7 presents our conclusions and directions for future work.

## 2. Related Work

SH technology aims to support people in having a better quality of life. SH technology has been applied for many purposes like energy saving, security and safety, fall detection, light management, smoke and fire detection etc. SH is a society focused application of IoT [6], which bundles different technologies (e.g., sensor hardware/firmware, semantic, cloud, data modeling, reasoning, processing, communication technologies) together to build its vision [7]. There are often different structured components in layered architecture incorporated in a SH system. Those are the physical layer, communication layer, and processing layer. Data is collected as the physical layer by sensors, transmitted through the communication layer to the processing unit in the processing layer where it is analyzed for activity recognition and behavior patterns discovery. The outcome of the analysis in the form of specific information, alerts or warnings may be communicated through the interface layer to various stakeholders (resident, caregivers, resident’s relatives) [4]. For SH application, challenges exist in each layer which can be summarized as:

- Data acquisition and communication;
- Data process and context aware;
- Context reasoning and decision support.

In past periods, sensor technologies, especially low-power, low-cost, high-capacity, and miniaturized sensors, wired and wireless communication networks have advanced the technology in SH research [8,9,10,11,12,13,14,15,16]. With raw data which are captured by these sensors, further contextual information of SHs could be generated by processing raw sensor data. Modeling, reasoning, and distribution of context in relation to sensor data play a critical role in SH challenges [7]. Context-aware computing has been proven to be successful in understanding sensor data, and auto acquiring and understanding the contextual information makes it easier to perform machine communication and enables further context reasoning. Therefore, context acquisition and reasoning about entities in SH has gained more interest among researchers in ubiquitous computing [17]. For example, sensor-based ambient-assisted living (AAL) research aims to exploit activity monitoring, recognition, and assistance to support independent living and ageing in place [11,18,19,20,21].

With the help of those researches about context computing based on sensor data, different demand-based services for SH are possible. The goal of supporting people to have a better quality of life and ensuring elderly can live comfortably and independently can be realized with the help of these SH technologies [12]. With favorable findings on their effectiveness, smart home technologies have enabled continuous monitoring, improved psycho-social benefits and enhanced the overall sense of well-being for users [22,23,24,25]. Tuan Anh Nguyen et al. proposed an activity recognition solution that effectively handles multiple-user, multiple-area situations, rapidly recognizing office activities as inputs for building energy and comfort management systems. The study represents a good effort at furthering applications based on activity recognition [26]. Alexander G.L et al proposed an early illness warning system consisting of algorithms which analyzed resident activity patterns obtained from sensors embedded in residents’ apartments. They designed an automated reasoning system to generate clinically relevant alerts which are sent to clinicians when significant changes occur in the sensor data, for example, declining activity levels [27]. It consists of algorithms which analyze resident activity patterns obtained from sensors embedded in residents’ apartments, but the knowledge expression of the reasoning process from activity recognition to early illness detection is limited, and the decision making method for alerts is simple.

Safety assistance in daily life is another important function of SH. Some SH systems serve as reminder systems for safety purposes. Some examples of the tasks that are performed for the resident are turning the stove off, stopping the running of bath water, and locking doors [28]. Many of these reminder systems perform the task if the resident does not respond, in order to ensure resident safety. However, the risk recognition in daily life may be a complex process based on contextual information. Risk in the home may be classified according to different degrees (discussed in Section 4) and may be updated with time lapses. How to reason different degrees of risk based on existing contextual information, and how to perceive changes in levels of risk with time lapses have not been discussed in previous studies.

For another, the simple reminder service in single SHs is not sufficient for providing safety services for SHs. The home is not only a single unit but also a component of a neighborhood under centralized management. In [29], an interactive health care system is proposed, aiming at enabling interaction between persons under care and incorporating the system in various living spaces, as based on motion-sensing interactions. Accordingly, flexible interactive services should be provided by different service providers. Therefore, safety service technology in SH management systems comprises not only integration of sensor technology, electrical equipment automation, wireless network technology, activity modeling and pattern recognition, but also needs further SH context reasoning and adaptation, and further decision support for service provision. However, thus far, the functions of SH mentioned in previous studies, particularly flexible safety services, are seldom discussed or examined. Therefore, the focus of this contribution of our study is to explore a context aware and reasoning approach for risk recognition in SH that enables proper decision support for flexible safety services.

Knowledge-driven modeling and recognition had intended to make use of rich domain knowledge and heuristics for SH technology [11]. Ontology-based modeling and representation have been applied to SH. Wongpatikaseree et al. introduced a context aware activity recognition system. An ontology was exploited to model the context which is obtained through a set of sensors [30]. Studies showed that ontological techniques underperform the data-driven techniques like Hidden Markov Model (HMM) in the absence of temporal reasoning. However, when ontological techniques were extended with temporal information, their effectiveness became comparable to HMM. Ihn-Han Bae presents a method for Recognition of Activities of Daily Living (RADL) in sensor equipped SH [17]. Our work follows the research of Ihn-Han Bae et al. and Liming Chen et al. [17,18], but extends the risk recognition function and, accordingly, decision support function for service provision. The ontology structure has similarity in expression of device, person and status. The description of sensors and observations keeps with the format of the SSN ontology proposed by W3C [31,32]. However, our study pays more attention to event expression from the perspective of safety management. For example, more detailed expression of person class and property (stranger, adult, child, older, disable), more suitable service classification (massage service, auto service, manual service) from the perspective of neighborhood management, and event expression from the perspective of risk are incorporated. The proposed ontology focuses more on risk recognition and is service oriented. A novel approach is proposed for gaining new knowledge about risk recognition which is represented in SWRL rules and is directly mapped to the ontology. The ontology is knowledge-based and combined with the developed rules to extend the information inferred by the semantic framework, and form a decision support system for SH management in neighborhoods. More flexible service could be provided with the help of a decision support system.

## 3. Physical and Methodological Realization of the SH Management System

For safety service purposes, the SH management requirements are as follows. (1) Data processing and context acquisition capability: Numerous sensors located in home units of a neighbourhood generate a huge amount of data for processing, in which the home unit is a single department within buildings of a neighbourhood. It may contain different functions of rooms in which some devices and persons may be located. There are usually lots of home units in a neighbourhood; (2) Self-management capability: Management is difficult because tasks are scattered. Thus, the system must have self-management capability with decision support; (3) Flexibility in service scheduling and execution: depending on the status of the SH and the monitoring of the risk, the management system should be able to plan how to proceed. For example, suitable service provision according to the risk event and re-plan ability with the changing situation; (4) The system must be suitable for integrating a pervasive computing environment. To meet service requirements from a safety perspective, our study focuses on responding to the following needs:

- Clear expression of the risk situation in daily life without any ambiguity;
- Accurate contextual reasoning about temporary potential risk based on knowledge;
- Dynamic decision support for safety services;
- Accuracy in task scheduling and execution.

**Figure 1:**
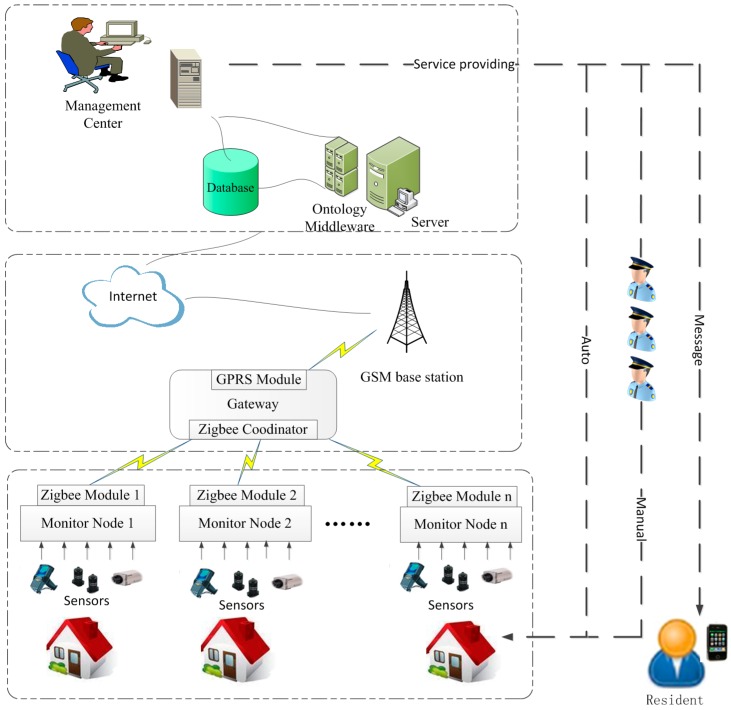
Functional description of the WSN-based SH management system.

We design an ontology-based SH safety management system that identifies and monitors risks in SH and supports decision-making. The overall architecture of the system is shown in Figure 1.

### 3.1. The Framework of the SH Management System

WSNs have been extensively used for environmental monitoring, forest fire prevention and military applications. A WSN is a self-organised wireless network that consists of numerous sensors. WSN nodes typically use an independent power supply, and thus, they can be easily deployed in large-scale and complex environments. Therefore, a WSN in an integrated framework for monitoring and controlling home environment is proposed and implemented to provide a reliable solution for SHs.

Unlike a common SH, which is an independent unit, each home unit is a centralised management unit in our system. Thus, each home unit is a component that transfers information to the manager, and the received intelligent safety service forms a different hierarchy. Figure 1 shows that the system is composed of three layers. (1) Sensing and actuating: Each home unit is a monitoring node distributed in a system; it is connected to a sufficient number of sensors. A home contains device sensors, contact sensors, position sensors and cameras. Device sensors detect if electronic devices have been turned on or off. Contact sensors are attached to the entry door, containers of teabag, sugar, milk, coffee and chocolate, etc. [18]. The activation of a contact sensor indicates the occurrence of an action involving the object to which the sensor is attached. Position sensors are placed in each room to monitor the movement of a person throughout the home environment, and cameras are used to identify the residents of a house (e.g., father, mother, children or stranger) [17]. Some actuators are deployed in home to control the on-off state of electrical devices. The actuators are some remote control switches series connected with power supply of electrical devices. Therefore the power of electrical devices could be remote controlled through wireless network; (2) Network: WSN has been used most to implement a smart home control network [1]. For the merit of WSN which is easily deployed in large area for real-time online monitoring, it is very suits for the large members of SHs’ management in neighbourhood. But there are large amount of sensed data need to be processed which would exhaust so much energy. Mingfu Li et al. used WSN and power line communications (PLCs) to reduce the unnecessary energy consumption of a smart home [33]. Zucheng Huang et al. adopt 6LoWPAN which is an IP-based communication standard for WSN instead of PLC to get better performance in transmission rate, signal coverage range, compatibility and extensibility [34]. Recently, ZigBee technology, which has exhibited the merits of low energy, short transmit distance, low cost and low complexity, was adopted as a good wireless solution for WSN [35,36]. In our system, sensed data are aggregated to the gateway node through ZigBee wireless protocols and sent to the remote centre server via general packet radio service (GPRS) modules that function as gateway nodes; (3) Application and decision: The remote centre server received the communicated data. An ontology middleware is modularized in the server to perform contextual information acquisition and provides reasoning and decision-making support. The framework of ontology is mapped to a database which stores the data and the ontological relationships. With the help of ontology middleware, the management centre could make decisions and provide safety services.

### 3.2. Ontology Model for the System

Ontological modelling is the process to (1) explicitly specify key concepts and the relationships among them for a problem domain and (2) build a hierarchical structure to encode the concepts and their interrelations using the commonly shared terms in the problem domain. The resulting ontologies are essentially shared knowledge models that enhance the capabilities of automated processing and the level of automation by allowing machines or agents to interpret data/information and reason against ontological contexts, thus enabling knowledge based intelligent decision support [18]. In our system, the semantic web ontology is used to represent the temporal contexts. Through context information modelling, ontology language is used and the SH management system can share knowledge about contexts among the other objects and can explain the contextual knowledge [37].

**Figure 2:**
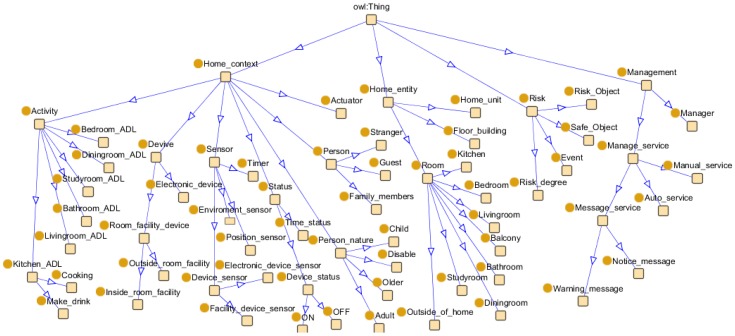
Top-level view of SH management ontology.

From the perspective of the safety management of SHs in a neighbourhood, we focus on the knowledge expression of SHs. The description of knowledge in SHs is clearly shown and contains specifications of domestic elements. Ontologies for real-world applications are complex and should be modularised [38]. The top view of the designed ontology is shown in Figure 2.

To meet safety management purposes, the ontology is described from the perspective of home context, activity, risk, and service, which is designed to express key concepts and relationships, divided into conceptual modules.

1. A home context perspective. With a focus on contextual information about SH, home context includes devices and their monitoring sensors, residents and their current state (on, off, time interval). Their relationships are defined, for instance, residents “locate_in”room.
2. Person activity. Activities are the explicit representation of a hierarchy of activities that consists of activity types and their relationships in a problem domain. Activities in ontologies are modeled not only based on objects, environmental elements and events but also the interrelationships between them, such as “is_a” or “part_of” relationships [18].
3. Risk perspective, with a focus on the elements related to a risk situation (object, event, condition) and classifies the degree of risk.
4. Service perspective, with a focus on the services provided (who provides the service, who receives the service and what is the content of the service).

**Figure 3:**
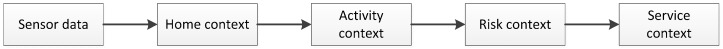
The relationships between each module in the SH management ontology.

The relationships between each module in the ontology are shown in Figure 3. Sensor data are mapped to the ontology and form formatted home contextual information with semantic knowledge. Activity recognition is performed using individually preferred algorithms and forms the activity context. Risk context can be obtained on the basis of home context and activity context. Finally, service context is generated for safety service purpose. With the help of the ontology, this facilitates interoperability and integration in terms of the shared structure and terminology. These features make ontological modeling increasingly popular for SH to provide automatic cognitive assistance.

The details of the structure of the ontology model and the relationships between its properties are discussed in the following sections.

#### 3.2.1. Home Context Perspective

**Figure 4:**
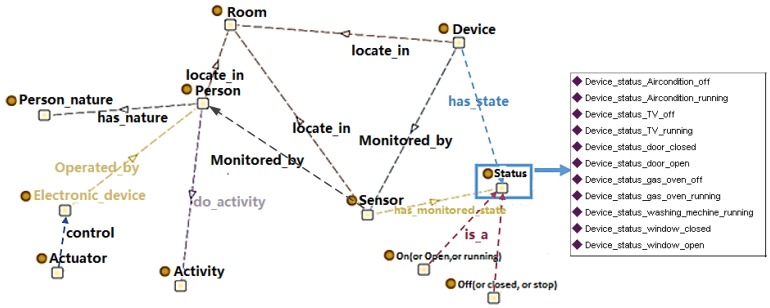
Ontology view focusing on home context knowledge.

An SH management system is responsible for many home units. Each home unit is a separated SH. We construct the ontology from the perspective of home context. The structure of the ontology is shown in Figure 4. Properties are used to link the individuals of the classes to explain the relationships between these individuals. Part of the properties are shown in Table 1.

For the perspective of home context, the knowledge of the SH ontology is described according to the following aspects.

1. Sensors and their monitored device: The “Device” class, the “Sensor” class and the “Actuator” class are defined as a subclass of “Home_context”. We learn some expression pattern of sensors for measurement processes, observations and deployment in SSN ontology [31]. The “Device” class expresses the types of device that are used at home. We classify devices as electrical devices, which include electrical equipment at home, such as TV, washing machine, air conditioner or microwave oven; facility devices, which include other types of facilities at home which are not electrically driven, such as sofa, bed, windows and doors; and unfixed facilities, such as bed and soft, or cup and bowl. The “Sensor” class defines various sensors that are used to “sense” devices at home. It monitors the state of devices, such as on/off (electrical device) or open/closed (facility device). Operating time is monitored by timer sensors. Thus, a property “monitored_by” is defined to link the “Device” class and the “Sensor” class. The “Actuator” class is used to define the actuators which could control the running state of electronic device. It is linked to “Electronic_device” class with an “acting” property.
2. Person and person’s attribute: The “Person” class is used to identify the person at home. It defines a person as a family member, a guest or a stranger. The “Person_nature” class is used to define the characteristics of different persons. It has subclasses, namely, “Adult”, “Child”, “Older” and “Disabled”. The “has_nature” property links the “Person” class to the “Person_nature” class. One person may have multiple natures. For example, a person may be older and disabled. A datatype property “Person_information”, which has the subproperties “Name”, “Age”, “Sex” and “Tel”, is attached to the “Person” class.
3. State: We define the state as a snapshot of behaviour of a device or a person in SH at a specific time window. To indicate the monitored state, the “Status” class is defined, which is linked by the property “has_monitored_state” to the “Sensor” class and determines the sensing results. Several instances are created to describe the state of devices, such as “Device_status_door_closed” or “Device_status_gas_oven_running” (Figure 3). The “locate_ in” property links the “Person”, “Device” and “Sensor” classes to the “Room” class and indicates their position. The position of a person changes, which is monitored by position sensors.
4. Activity: The “Activity” class expresses the action of a person. The activity cannot be directly identified by sensors. However, an activity is related to contextual entities, e.g., person, location, objects, sensor observations, etc. Beside the instance mentioned in Section 3.2, some specific situations that correspond to an unknown activity could be reached by aggregating sensor observations along a time line.

#### 3.2.2. Activity Perspective

**Figure 5:**
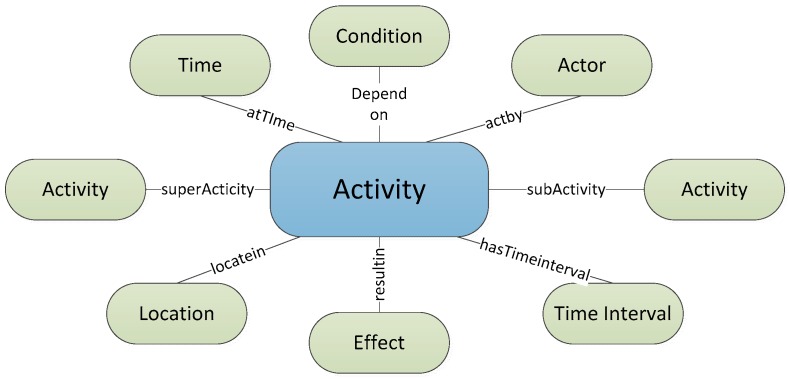
The concept model of activity.

Research on sensor-based activity recognition has recently made significant progress and is attracting growing attention in a number of disciplines and application domains [11]. A growing number of workshops have been dedicated to activity recognition research from different research angles and communities, in which a knowledge-driven approach is an effective method to make use of rich domain knowledge and heuristics for activity recognition in SH. Most ontologies are proposed for human behaviour recognition [2,17,39,40]. Similarly, contextual knowledge of the user, role, location, environment, time, context sources, and proper behaviour granularity levels are needed to conduct contextual reasoning. Our approach follows the method mentioned above to recognize the activity of a person in a SH. Events in daily life are related to human activity which can be described by a number of properties that relate to other physical objects and conceptual entities. As it can be seen from Figure 5, properties like time, location and actor represent the context within which the activity takes place. Properties such as conditions and effects represent the causal and/or functional relations that are used for inference during activity level reasoning. Subclass and superclass properties denote the type and inter relationship between activities.

#### 3.2.3. Risk Perspective

**Figure 6:**
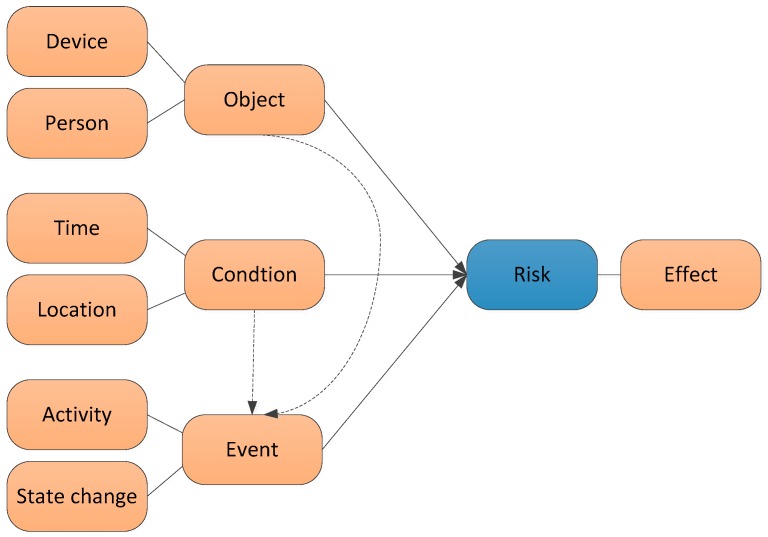
The concept model of risk events.

The recognition of disk should be based on some pre-condition. We define them as object, condition and event, as shown in Figure 6. Object is the subject of risk. It includes device and person such as oven or child. However, not all devices are risk objects, for example, adult residents or sofa, which are not considered as posing risk. The event is the activity or change in state of the object which should be recognized based on the context of the object and condition. Those events alone may not have risk, but when occurring together and under some conditions. Therefore, we could define a risk situation is an accumulation of states of specific events which is related with specific objects in a particular time window. However, not all the situations are risk situations, they should be recognized by a further reasoning process.

**Figure 7:**
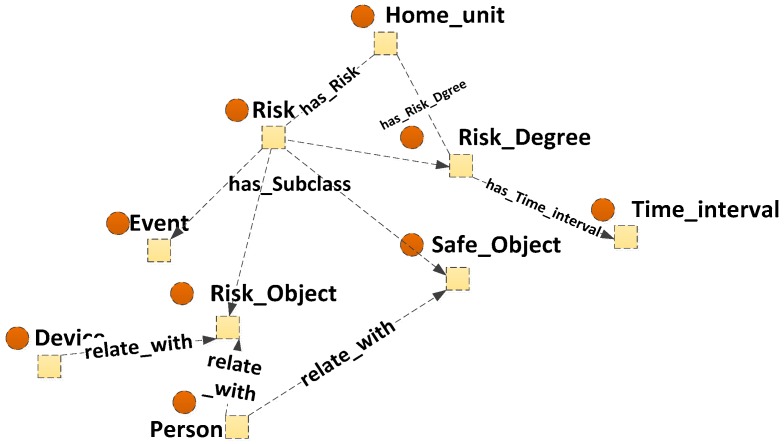
Ontology view focusing on risk knowledge.

To express the contextual knowledge about risk, the ontology is constructed from the perspective of risk context. As shown in Figure 7, a “Risk” class is built to express the knowledge which is related with risk event. It has subclasses of “Risk_Object”, “Event”, “Safe_Object” and a “Risk_Degree” subclass is used to expresses the degree of severity of a risk event. For “Risk_Object”, “Event”, “Safe_Object” classes, there are no subclasses in it, but linked by a “relate_with” property to related classes. Some instances are linked to it by predefinition. For example, an instance of “Person” which relates with nature of “Child” in “Person_nature” class, related with (“relate_with”) “Risk_Object”. The instances of “Device” class which are considered as possibly leading to risk are linked to “Risk_Object” with “is_a” property. The “Event” class also linked with some events may lead to risk, for example, “cooking” activity. A “has_Risk”property links the “Home_unit” class to “Risk” for indicating whether the risk event occurs in the SH or not. A “has_Risk_Degree” property links the “Home_unit” class to “Risk_Dgree” for recording the degree of risk event in a SH. If a risk is recognized, its extent will be evaluated. The instance of “Time_interval” class which is linked to “Risk_Dgree” by “has_Time_interval” property records the temporary state of risk. Table 2 shows some properties’ relationships between relevant classes of risk.

#### 3.2.4. Service Perspective

**Figure 8:**
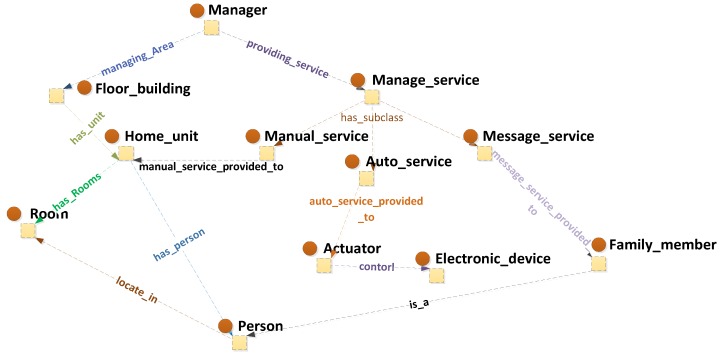
Ontology view focusing on service knowledge.

We construct the ontology also from the perspective of service. Before providing services, some questions should be clarified. Who needs to be serviced? Who provides the service? What types of services should be provided? To address these questions clearly, the “Management” class is defined in our ontology, as shown in Figure 8. It has two subclasses, namely, “Manager” and “Manage_service”. “Manager” is the service provider. Numbers of manager instances are inserted and linked to the instances of the “Home_entity” class by the property “managing_Area”. Number of managers is responsible for the safety service of different home units in the neighbourhood. The “Home_entity” class, which includes the subclasses “Floor_building”, “Home_unit” and “Room”, defines the precise location of home units and rooms based on their properties, i.e., “has_unit” and “has_rooms”. The “Manage_ service” class, which has three subclasses, namely, “Auto service”, “Manual service” and “Message service”, is defined as the service type. “Message service” is divided into “Notice_message” and “Warning_message”. The “Manager” class is linked to the “Manage_service” class and determines who should provide the service. A property “provide_service_to” with three sub-properties links the “Manage_ service” class to the “Home_unit” class, the “Family_member” class or the “Actuator” class and identifies who will receive the service. Figure 5 illustrates the property relations of the service provided. Table 3 shows some property relationships between relevant classes of service.

## 4. Reasoning Method

The system aims to detect events which influence safety, and subsequently, provides safety service to residents in SHs. Therefore, the reasoning ability is important for the system. With raw data captured by the system, different stages of reasoning are performed.

(1) Contextual information awareness: the raw data of SH are formed as formatted contextual information with semantic knowledge which is defined as low level context.
(2) Daily activity recognition based on basic contextual information.
(3) Risk detected and associated to a risk degree.
(4) Decision support for the service provision (such as send an alert about the risk event) and updated service with the degree of risk of the detected risk.
(5) Steps repeated until the risk detected has been eliminated.

In our work, ontology has enabled modelling the context of the Smart Home in a formal way and to proceed to inference. The relations in the SH domain are defined in ontology. However, although the proposed ontology has provided basic contextual knowledge of SHs and was used in the memory of the server, additional rules still should be established to extend the information inferred by the semantic framework. There are some implicit knowledge and implicit relations between SH environment and daily risk event. On their own, OWL-DL ontologies are not sufficiently expressive to specify reasoning rules. As the ontology describes the relationships between resources of a specific domain, the reasoner could reason implicit unknown relations from known relations with the help of certain rules. The hierarchical relationships of the semantic web was first proposed by Berners-Lee [41]. The reasoning rule which is constructed based on ontology and DL made the knowledge expression and reasoning possible. SWRL (Semantic Web Rule Language) which was developed by Stanford University is a rule language that is highly integrated with Protégé. It complements the definition of the rule based on OWL, and composites OWL DL, OWL Lite, and unitary and binary Datalog RuleML language. Similar to OWL, SWRL can be used to establish the rules to explain the OWL individuals and infer new knowledge about these individuals [42]. Therefore, in our study, the inference rules are commonly specified by means of SWRL on the basis of a semantic description in ontology. We choose Jess (Java Expert System Shell) as the semantic reasoner for reasoning. A Jess engine running inside the Protégé framework is the basis for the JessTab integration model. In Protege-OWL, a SWRLJessTab is a plug-in to the SWRLTab that supports the execution of SWRL rules using the Jess rule engine. It provides a graphical interface to interact with the SWRLJessBridge.

**Figure 9:**
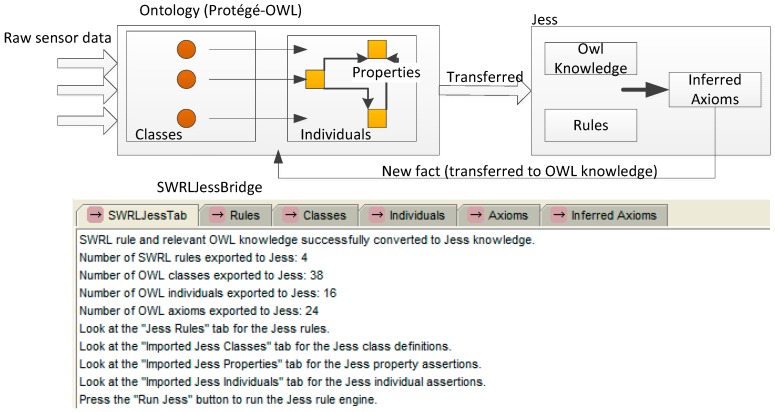
Reasoning process with the help of Protege-OWL/Jess.

As shown in Figure 9, in our system, a large amount of raw data are collected by the sensors located in each room of every SH, include signals form device sensors, contact sensors, position sensors and cameras. The raw data are mapped to the individuals in ontology to be transferred to contextual information with semantic knowledge. The temporal contextual information could indicate the state of SH at a specific time window. In each time window, the state of the current time window is compared with the state of the previous time window. If any changes are found, then the inference is performed immediately. The SWRL rule and relevant OWL knowledge are converted to Jess knowledge. With relevant rules, new facts are inferred. These facts may be some activities undertaken by the person, such as cooking. The new Jess facts are recorded in the knowledge base and transferred back to Protégé-OWL as OWL knowledge. We define the context about new facts as high level context which may be a recognized activity or a risk situation and transferred back to the ontology for further reasoning. In the subsequent time windows, the comparing and inferring is continued. If a risk situation is inferred, the service decision process is invoked. The risk event and degree are recorded as new facts and transferred back to Protégé-OWL. The reasoning is continued and repeated to detect any variances in the risk situation.

The reasoning content mainly includes daily activity recognition, risk situation recognition, and decision support for service provision.

### 4.1. Daily Activity Recognition

An activity at a specific time window can be described as the accumulation of states which occurred within that particular time window. Let us take a simple example to explain this. Suppose that the resident Yan moves to the kitchen, which is detected at 10:00 a.m., and the contact sensor attached to a cup is activated. After that, sensor data about use of coffee is obtained. A “30 SEC” time duration is record by the timer, and is mapped to the linguistic contextual data “M (medium)” by a fuzzy membership function for the time duration following the method proposed by Ihn-Han Bae [17]. By matching this situation against activity ontologies, the activity class that mostly overlaps with the situation (e.g., “Make_Coffee”) is considered to be the actual activity. Then, we can infer that the person Yan is going to make a coffee [18].

In our system, as the conceptual models have been structured and represented in the SH ontology, if a number of properties defined by the ontology are observed, and linked to form a description of a specific context, the unknown activity described by the perceived properties can then be inferred through descriptive reasoning against the SH ontology. Let us take “cooking” activity recognition as an example. To support the use of ontologies for activity recognition, context ontologies are required to conceptualise contextual entities formally, e.g., time, location, objects, sensor observations, and their relationships, as shown in Table 4, whereby “Time” and “Time_interval” indicate the temporal references to the activity.

**Figure 10:**
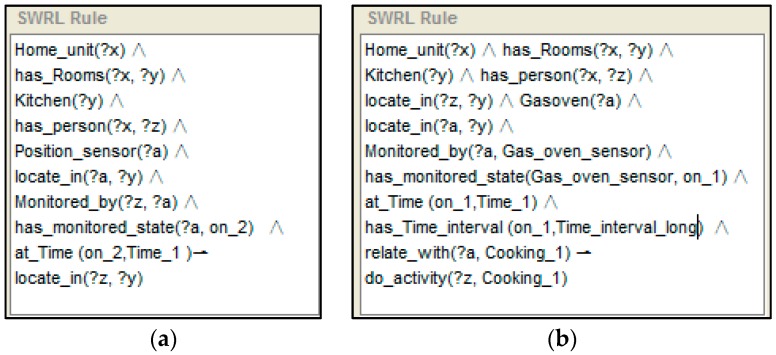
An example of person activity recognition reasoning based on a rule (cooking, in this case). The first one (**a**) shows the recognition of the position of a person. The second one (**b**) shows the recognition of the activity.

Let us take an example to express the reasoning process with the help of the SWRL rule, which is shown in Figure 7. A SWRL rule may be defined as a rule that, if all the atoms in the antecedent are true, then the consequent results must also be true. In SWRL rules, the symbol “^” represents a conjunction, “?x” denotes a variable and “!” indicates the implication. If “?” does not exist in the variable, then an individual is present. With captured temporal context information expression of the ontology, reasoning follows the rule. A situation using stored knowledge (e.g., “a home unit exists, this home unit has a kitchen and has a person, and the kitchen has a gas oven”) is defined first, and then expresses a state for it. The rule shown to the left of Figure 10 is the recognition of the position of a person. As the person “z” is detected by the position sensor “a” which is located in kitchen “y”, the reasoning engine inferred that person “z” is locate in kitchen “y”. The rule which is shown to the right of Figure 7 expresses the state which includes dropping a particular triple (In a same time window, the person is detected to “located” in the kitchen, and the gas oven sensor is detected has state “On” with “Time_interval_long”). Kitchen is related with a generic activity: “Kitchen ADL”. Its descendants “cooking” and “making drink” can be specific activities. Also, the “gas oven” is related with “cooking”. Hence, an activity is inferred (person “z” who is located in the kitchen of home unit “x” is now cooking).

### 4.2. Risk Situation Recognition

Based on low level and high level contexts, which are mentioned above, recognition and assessment of danger is possible. To perform risk recognition, a reasoning method is proposed to infer new information from the contextual information and take advantage of the implicit rules of relationships among concepts. The risk recognition algorithm in our approach can be described as follows:

- A risk object set OR {OR_1_, OR_2_, OR_3_... OR_n_} is built. For some devices or a person who may be at risk, we defined them as the risk object. For example, a child is playing may drop from a window, or an oven left on may lead to a fire, in which, child and oven are objects which may lead to risk. They are served as the elements, and added to OR. We define a “relate_with” property which links those subclasses to “Risk_object” class in ontology previous, as shown in Figure 4. However, this does not mean those objects are really risk objects but only some common object in daily risk situations. Only under some specific conditions may they lead to risk. Most of the time, they are safe, such as when the oven is not running.
- Another set of events ER {ER_1_, ER_2_, ER_3_... ER_n_} is built which contains the event elements related with the elements in set OR. We define the event as an activity or a state of person or device. For example, the activity “cooking” is an event, and the state change of “running” of oven is also an event, if the event in the set ER is aware of the object in OR, such as an oven that is running. It is a condition which may lead to risk, but not necessarily. When an event occurs in a risk situation and is related with risk, it is a risk event.
- The situation is conceptualised as a snapshot of states at a specific time window in a physical or conceptual environment [17]. It may contain one event or several events occurring in one same time window as well as a detailed description of events which are related to a person, object or device object and the time window it take places. For example, in a time window, the mother is detected as being located in the kitchen, and the state of oven is detected as “running” for a “Medium” time. The situation which involves risk is a risk situation.
- To assess whether the situation has become a risk situation, another object set PS {PS_1_, PS_2_, PS_3_... PS_n_} is built, which represents the person not at risk, such as the adult resident. The state of PS_i_ is checked. If it has some same states with OR_i_, the situation is recognized as safe. For example, after a “cooking” activity which is related with “Oven” has been recognised, if an adult resident is aware and has been detected as “located in kitchen” in the same time window, the situation is recognized as safe, otherwise, the situation is continually monitored.
- The change detection compares the context situation of the current time window τ(i) with the context situation of the previous time window τ(i–1). For the second condition in step d, if the current context situation differs from the previous context situation, for example, the adult person is detected back to kitchen (locate in kitchen), then the event is still safe, otherwise, the event is recognised as “Risk”.
- The comparison is performed continuously in each time window to monitor risk until the “cooking” event is detected as being finished.

**Figure 11:**
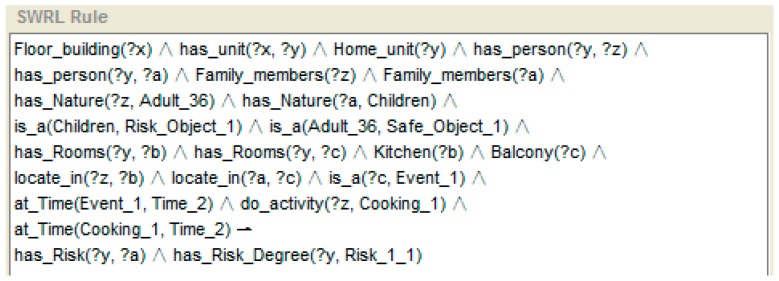
An example of risk recognition reasoning based on rule (child on balcony along).

An example of risk recognition reasoning based on SWRL rule is shown in Figure 11. This example shows a further risk reasoning process which is on the basis of the recognition of “cooking” activity of the person “z” as shown in Figure 10. The time interval and the state of the gas oven show that the cooking activity has not ended. The adult person “z” is still in the kitchen. Also, a child “a” is detected as being located on the balcony. According to the proposed approach mentioned in Section 3.2.3, the person who has nature of “Child” is classified as OR and “‘located_in’ ‘Balcony’” is classified as ER. When the child is detected as moving to the balcony, it means that an element in OR sets a linkage with ER. Then, this recognised condition may be a dangerous situation but not necessarily. In the same time window, the adult person who is classified as part of set OS is still located in the kitchen. No link is set up between OR and OS. Then, the risk is recognised. As the risk is first recognised, this situation is recognised as degree I. The inferred conclusion indicates that the risk event discovered at home unit “y”. The “has_Risk” property points the risk to child “a”. The “has_Risk_Degree” indicates the degree of risk.

### 4.3. Upgraded Risk Degree Detection

To classify the severity of different risks in daily life, four degrees of risk are addressed as shown in Table 5. Degree I indicates that a situation which slightly influences safety has occurred. For example, an electrical device that has been operating for too long time may cause danger. Degree II indicates a situation which will lead to a more serious danger than Degree I. For example, when a baby crawls onto the balcony without the supervision of adults, a warning message will be sent to the host. Degree III indicates that a dangerous situation must be dealt with immediately. Services that address such a situation can be acted upon by actuators installed in homes. For example, if a dangerous appliance is still operating when a resident leaves home, the safety service will control the actuator to shut off the appliance automatically. Degree IV indicates that a dangerous situation which cannot be addressed automatically by actuators has occurred. In this case, the manager must provide manual service. For example, a resident has left his/her home for a long time but forgot to lock the door.

**Figure 12:**
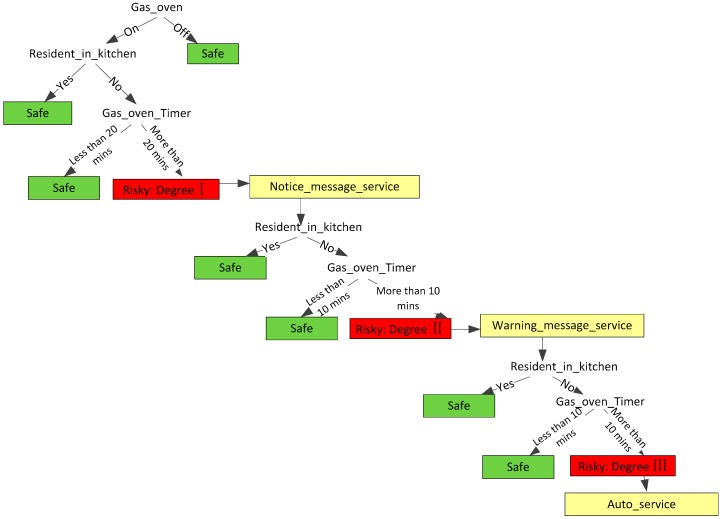
The Structure of Risk DT.

Over time, the degree of danger of a risk could be upgraded. For example, if a dangerous appliance has been operating for a long time but a resident does not act or respond to the manager centre when he/she receives a notice message, the degree of danger is upgraded. In order to make a contextual adaption of risk degree, a decision tree (DT) method is used. Many researchers applied decision trees to model events of daily living in a multi-resident context [4,37,43]. An example of DT used in our approach is shown in Figure 12 which shows a classification tree with classes: safe and risky (Degree I, Degree II, Degree III). The tree can be rewritten as a set of IF-THEN rules. For instance, the rule: IF “Resident_in_kitchen” = NO and “Gas_goven_Timer” > 20 THEN degree = “Risky degree I” and “Notice_message_service“ = Ture; IF “Notice_message_service” = Ture and “Resident_in_kitchen” = NO and “Gas_goven_Timer” > 10 THEN degree = “Risky degree II” and “Warning_message_servic” = Ture; IF “Warning_message_service” = Ture and “Resident_in_kitchen” = NO and “Gas_goven_Timer” > 10 THEN degree = “Risky degree III” and “Auto_message_service” = Ture. The case in Figure 12 also shows how the risk of a situation is upgraded with variations in environmental context (in this instance, this is resident state and time state).

**Figure 13:**
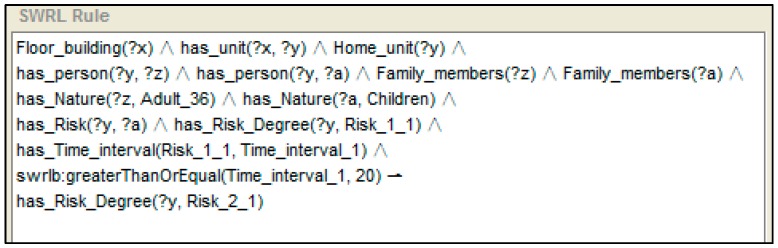
An example of reasoning for the upgrade of the degree of risk.

Another example of upgraded degree of risk reasoning according to instance shown in Figure 11 is presented in Figure 13. A “Time_interval” instance records the duration of the event “child located on balcony”. The “Time_interval” is related with the “Risk_1_1” by “has_Time_interval” property. Therefore, on the basis of the risk recognised above, if the situation lasts for more than 20 min [swrlb: moreThan (Time_interval, 20)], a degree II risk is recognised.

### 4.4. Decision Support for Service

Finally, for different risk events, appropriate service should be provided. All decisions about safety services are made automatically by the management centre according to the contextual reasoning. As shown in Table 1, the service for Degrees I and II is provided by the remote centre server, which sends notices or warning messages to residents. The service for Degree III is provided by actuators in SHs. The service for Degree IV is provided by managers of the neighbourhood who receive the service message from the remote centre server. However, some uncertainty still exists. This includes:

- Who needs to be serviced?
- Who provides the service?
- What types of services should be provided?

**Figure 14:**
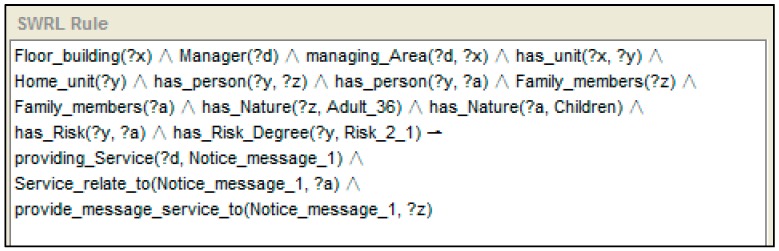
An example of service provision expressed by the SWRL rule.

We have developed a decision support process with the help of knowledge-based adaptions incorporating context awareness and reasoning about risk events. For this purpose, the ontology is designed from a service perspective in centrally managed neighbourhoods, and relevant reasoning rules are designed. According to the classification in Table 1, different degrees of service have different service receivers, different service manners and different service providers. The links has been defined in the ontology as expressed in Section 3.2.4. An example of service provision which is expressed in rules and follows the instance mentioned in Figure 14 is shown below. With the context of SH, and where risk expression has been reasoned, the “providing_Service” property indicates who should provide the service (“d” is a instance represent the remote server) and the type of service that should be provided (notice_message_1), “service_relate_to” indicates the content of the service (child “a”), and “provide_message_service_to” indicates who should receive the message service (adult “z”).

## 5. Integration of the Proposed Approach

The following steps indicate the work flow of the proposed approach. The steps shows how the ontology is combined with SWRL to process reasoning and make service decisions.

**(1) Extract relevant information from the context:** using on-board sensors and specific algorithms, the SH extracts relevant information from its environment. Sensors give information about detection in their line of sight. The state of the monitored objects will be compared with those of a previous situation. In the SH scenario, examples of such information are: device state (on, off...), person state (location, move...), environment state (temperature, humidity, and time…), etc. If the current situation differs from the previous situation, an event (not only a data) is published.

**(2) Instantiated ontology updated:** The relevant contextual information is extracted and mapped to the ontology with the help of relationship definitions given in the ontology. For example, when a position sensor detects someone, it publishes an event which is composed of its topic (sensor topic), the timestamp, the sensor value, the sensor unit (no unit for that kind of sensor), the confidence value, the sensor ID and the message ID [44].

**Figure 15:**
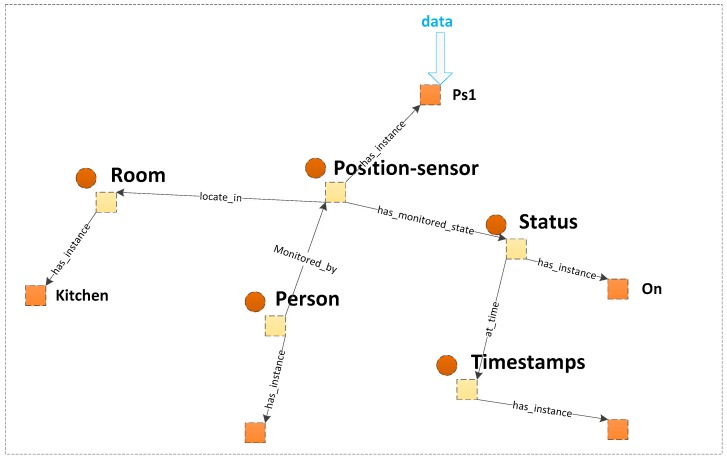
An example of the updated ontology with position sensor data.

When the event is sent, the services will fill the ontology with information about the event. To input the data to the right place in the ontology, this service will read the ontology to find the sensor in the ontology. When the right sensor is found, the service receives a different value in the ontology. With the possibility of there being a relationship between the individuals in the ontology, it is possible to have more information than just the sensor information. Figure 15 shows an example of the updated ontology with new data from the sensor position. With the sensor data, we can deduce other information (the person is detected by position sensor, the related sensor is located in the kitchen, kitchen is located in the home, kitchen is a room, and the kitchen becomes an occupied room, etc.).

**Figure 16:**
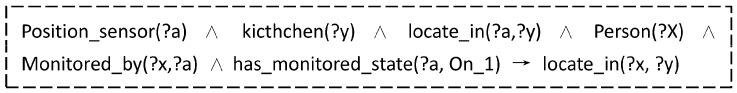
Location of person deduced by rule.

**(3) Check the rules and perform the reasoning:** After the ontology is filled with data, new contextual information can be deduced. Inference rules enable that. The reasoner, as soon as new information is set in the Triple Space, checks the rules written for the application to find which rules should be activated. For example, with an updated instance, the location of the person is deduced by the rule defined previously, as shown in Figure 16.

The reasoner executes the activated rules and the status of the instantiated ontology is updated again, reflecting the conclusions extracted by the reasoner. If the fillers of a number of properties are observed and linked to form a description, i.e., a specific context, the unknown activity described by the perceived properties can then be inferred through descriptive reasoning against the ontologies. Further, the unknown risk could be deduced.

**(4) Task establishment:** The appropriate safety service is suggested through intelligent decision making by the reasoning engine, and the service task is established.

**(5) Task execution:** The task is captured by the service management component, which acts consequently to provide appropriate service according to the suggestion of the reasoning engine. Then, the process repeats to check if there any risk in the SH.

**Figure 17:**
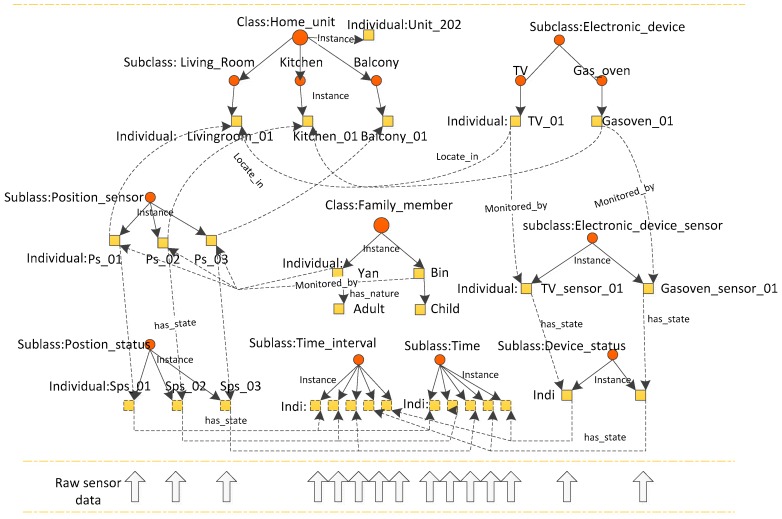
The updated knowledge base in the example.

The entire process involved in this approach is described using the succeeding example. A young mother and her three-year-old child are at home. The home unit has a living room, two bedrooms, a kitchen, and two balconies with several relevant devices. The home unit is located in a building in a neighbourhood. Manager Wang manages the floor the unit is located on. First, the ontology is instanced. The main relevant instances in this case are room individuals: “Livingroom_01”, “Kitchen_01” and “Balcony_01”; person individuals: “Yan”, “Bin”; electronic device: “TV_01”, “Gasoven_01”; position sensor individuals and electronic device individuals; individuals of sensor state and their time state individuals. In the ontology, properties are defined for all classes. After the instances are defined, the property relationships are linked to relevant instances. The instantiated ontology is shown in Figure 17.

With sensors deployed in the SH, the raw sensor data are mapped to the ontology. If the states are different, the relevant instances of the ontology are updated (Step 1).

**Figure 18:**
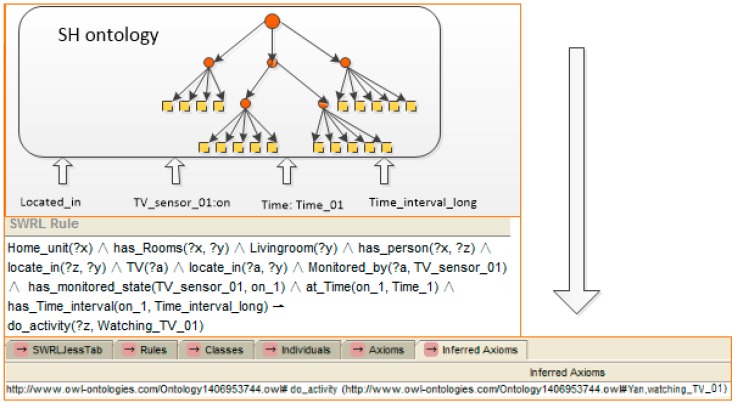
The reasoning process of activity ‘watching TV’.

In this case, at first, the young mother and her baby stay in the living room and watch TV. The positions of both persons and the state of the TV are monitored by the sensors. The property “locate_in” links the person instance “Mother” to the room instance “Livingroom”. The instance “Device_state”, which is linked to the instance “TV_sensor_01” by the property “monitored_by”, is updated with a new value: on (Step 2). The time duration of TV is mapped to the linguistic context data “(Long)”. The reasoner checks all the rules of the system. The rule shown in Figure 5 takes part in a part in the reasoning engine. The reasoning engine infers that the people at the home unit are watching TV (Step 3). The reasoning process is shown in Figure 18.

**Figure 19:**
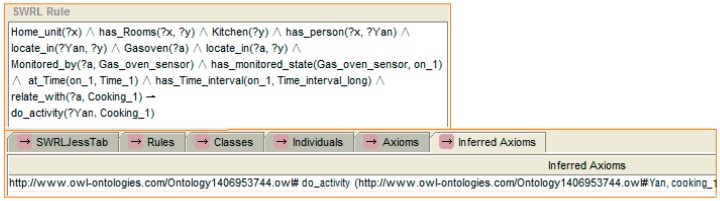
The reasoning process of activity “cooking”’.

After some time, the mother goes to the kitchen and turns on the oven. The action of the mother is “sensed” by the position sensor, and the state of the oven is “sensed” by the oven sensor. The ontology is updated with the new information. The time duration of the “ON” state of gas oven is mapped to “long” context. At the same time, the system compares the context situation of the current time window with that of a previous time window. If the situation are different, the reasoning engine performs the reasoning. According to the selected rules, the reasoning engine infers that the mother is cooking. The reasoning rule and result are shown in Figure 19.

**Figure 20:**
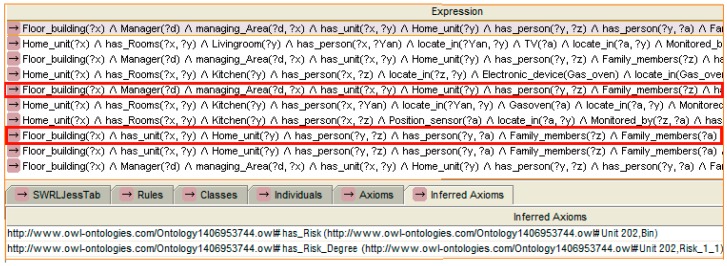
The rule selected and the reasoning result of the first risk.

After a few minutes, the baby goes to the balcony to play. The position sensor on the balcony detects the baby and generates a signal. The data are mapped to the ontology. Then, the new context information is checked by the comparing different time windows. The reasoning engine performs the reasoning. The reasoning result shows that the situation is recognised as a danger (Step 3). Figure 20 shows the rule selected and the reasoning result.

**Figure 21:**
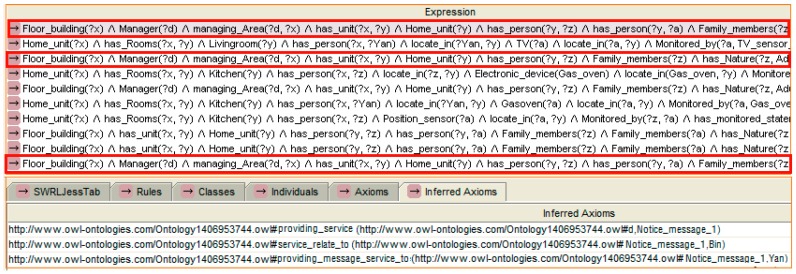
The rule selected and the reasoning result of the message service.

With the reasoning result, the reasoning engine checks relevant rules to make a decision regarding service provision. After that, a task is established by the service management component in the remote server (Step 4). According to the risk degree shown in Table 1, which indicates the rules, a notice message service is necessary. The service management component sends a notice message, which is the same as the instance value of “notice_message: child” given to the mother (Step 5). The reasoning result points out who should provide the service, who receives the message and what is the content of the risk. The reasoning rule selected and reasoning results are shown in Figure 21.

**Figure 22:**
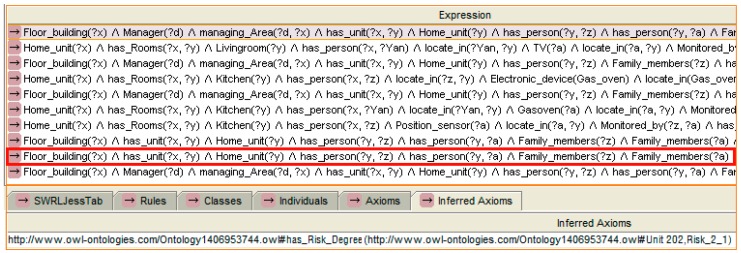
The rule selected and reasoning result of the risk update.

After the mother sees the message, she goes to the balcony to check on her baby. However, the mother forgets that the oven is still operating. After a few minutes (the time is monitored by a timer that checks the oven, and the threshold value can be modified by the user in the “data_type” property of the instance), the monitor node “senses” that the oven is still operating but nobody is located in the kitchen. The reasoning engine infers that a notice message should be sent. A new task is established. A notice message “Notice_message: oven” is sent to the mother. However, the mother is concentrating on taking care of her baby and does not listen to the phone. After a few minutes, the risk degree is updated. The reasoning rule selected and the reasoning results are shown in Figure 22.

According to the inference of the reasoning engine, a warning message “Warning_message: oven” is sent to the mother. If the mother still does not respond to the warning, then the risk degree continues to be updated. The remote control centre sends a command to the actuator of the oven. The oven is automatically turned off.

This example indicates the flow of information, the upgrading of the ontology, the activation of rules in the reasoning engine and the updating of a risk degree to provide appropriate safety service.

## 6. Evaluation

### 6.1. Experimental Setup

**Figure 23:**
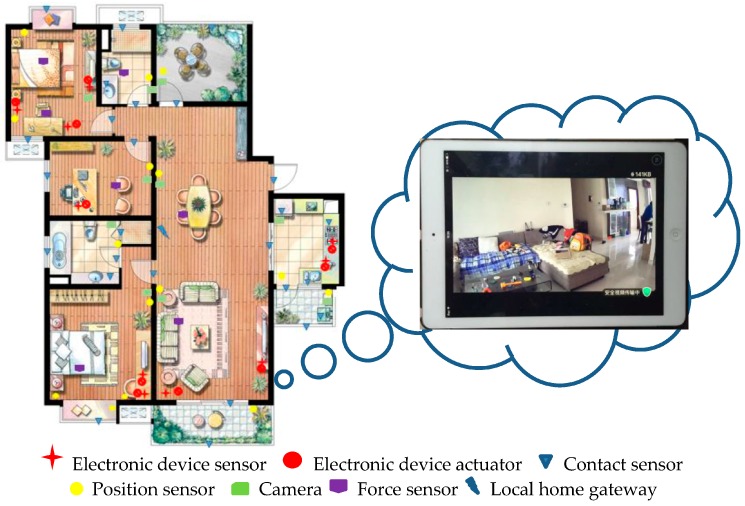
Layout of sensor deployment in the SH.

To evaluate and demonstrate the feasibility of the proposed approach in a real home, we perform the experiment in the neighbourhood of Jia Ri Feng Jing. Three apartments on the same floor of a building and which are the same type of housing are monitored. The layout of the SH with sensor location and placement is shown in Figure 23. Each test site, which is occupied by a group of candidates, includes two adult candidates and one child candidate, and consists of a living room, two bedrooms, one study room, one dining room, one kitchen, two washrooms and two balconies.

**Figure 24:**
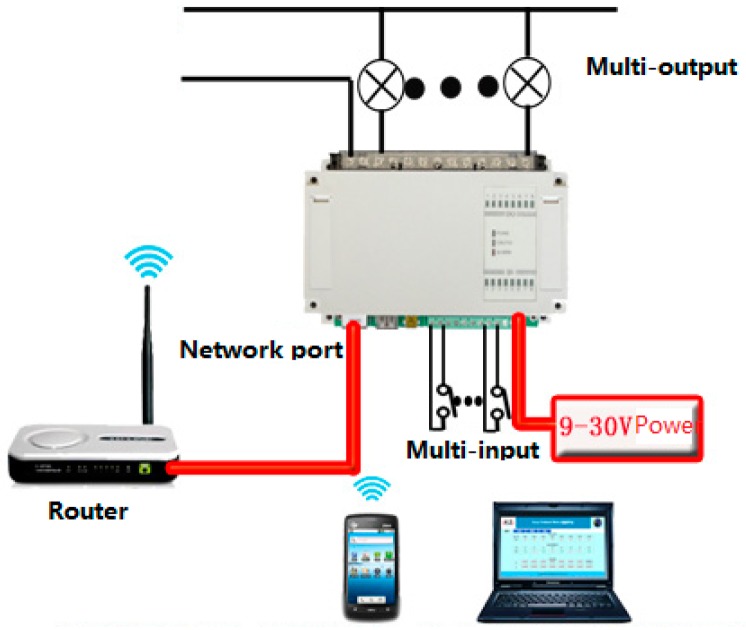
Remote control of the electronic device in SH.

A sensors network is deployed in the test site. There are a large amount of sensors including electronic device sensor (appliances sensing), contact sensor, position sensor, force sensor and camera. Electronic device sensors are added to the circuit of electronic device to detect if it has been turned on or off. Contact sensors are attached to the entry door, containers of teabag, sugar, milk, coffee and chocolate, etc. Position sensors are set in each room to detect the position and movement of a person. Force sensors are deployed in some home furniture such as the sofa, bed or a chair to detect a person’s exact location. A camera is used to distinguish different persons in a SH. To realise the remote control of the on/off state of electronic devices, the circuit is modified and linked to some relays which could be remotely controlled through an embedded web control device as shown in Figure 24. The electronic devices in SH could be remotely controlled through wireless internet.

The sampling algorithm of the sensor network is designed in such a way that it only sends the data to the remote server when there is a change. Otherwise, it sends it every 10 min. Adding time thresholds has a different significance for each type of sensor node but one common benefit is that, by this, the system identifies the dead and malfunctioning nodes in the network.

A computer is used as the remote server. The data from local end-nodes is transmitted to a coordinator connected in a home gateway. Then, a data packet is transmitted to the remote server through a base station. The data is processed and relevant contextual information is extracted by the ontology middleware in the remote server. The local SH only collects and communicates data. The task of analysing information to determine daily living activities and context-aware scenarios is performed by the remote server. Processed data is uploaded to a database which is directly mapped to the ontology. This is suitable for the centralised management of SHs in a large neighbourhood. Service tasks such as assigning a manager to provide manual service could be set up and performed immediately.

The experiments concern the recognition of risk situations simulated by three groups of candidates who perform relevant activities. For example, a candidate opens the oven and leaves the kitchen without anybody in it. In this case, the candidate should perform the activity of cooking and leave the kitchen. The situation is simulated respectively by three groups of candidates. Each situation is performed on schedule. Some typical cases (but not all) are listed in Table 6. A total of 68 cases were simulated. The risk types and service types are shown in Table 7. These cases include three types. The first type is about electronic device events such as an air-conditioning unit or a TV operating when nobody is at home, etc. Total number of this type of cases which are effectively performed is 28 (excludes the tests which may be invalid by faulty operation of the candidates). The second type is about person events such as a baby climbing up to a windowsill alone, etc. Total number of such cases which are effectively performed is 22 (excludes the invalid tests). The third type is about facility device events such as a person away from home for a long time but who forgot to close the door, etc. Total number of such cases which are effectively performed is 18 (excludes the invalid tests). The experiment was performed from morning (8:00) to afternoon (18:00) for seven days. A recorder records the experimental results. After each case simulation, the accuracy of the risk recognition result and relevant service decision are evaluated and recorded. The accuracy of recognition results of activities performed are also evaluated and recorded.

### 6.2. Experimental Result and Discussion

#### 6.2.1. Accuracy of the Risk Recognition

The sensor data were fed to the system prototype as if the sensor activation occurred in real time. When the data is played back, the system attempts to identify the degree of safety of an on-going event. All the events listed in Table 3 were played back in real time and processed by the system prototype for decision support.

We define the accuracy rate as the ratio of the accurately recognised cases to all successfully performed cases.

The accuracy rate of the activity recognition could be calculated by:

(1) $$A_{a}=1-\frac{\sum F_{a}}{AL_{a}}$$

in which, $A_{a}$ is the accuracy rate of the activity recognition, $\sum F_{a}$ is the falsely recognised activities and $AL_{a}$ is all activities performed.

The accuracy rate of the risk recognition could be calculated by:

(2) $$A_{r}=1-\frac{\sum F_{r}}{AL_{r}}$$

in which, $A_{r}$ is the accuracy rate of the risk recognition, $\sum F_{r}$ is the falsely recognised risks and $AL_{a}$ is all risk situation simulated.

**Figure 25:**
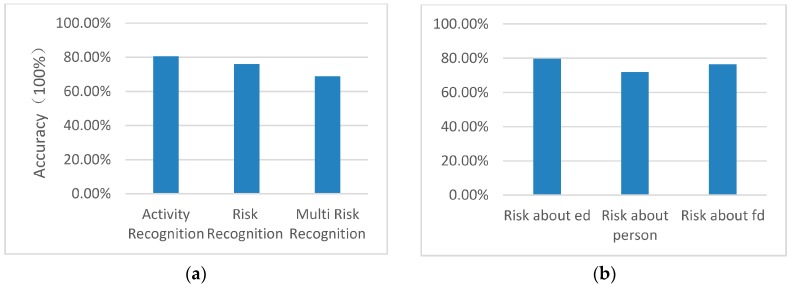
Accuracy rate of the experimental results. The first one (**a**) shows the accuracies of activity recognition, risk recognition and multi risk recognition. The second one (**b**) shows the accuracies of different types of risk (about ed, person and fd)

Figure 25 shows the results of the experiment. The accuracy rate of activity recognition is 80.51% which is not much different to similar research about ontology-based activity recognition (80.3% accuracy rate) and HMM (79.4% accuracy rate) [4]. However, considering the experiment was performed in multiple SHs and not a single SH, the results are still quite encouraging. The accuracy of risk recognition is 76.47%. As the risk recognition is based on activity recognition, this result is acceptable. When multiple risks take place in the same time window, the accuracy rate drops to 68.73%. This can be attributed to the transitions between events and how well the system keeps track of the recognised activities. We believe that this can be increased by using feedback from composite activity recognition segments. The accuracy rate of different types of risk events is still computed. As shown in Figure 11b, the accuracy rate of risk recognition of electronic devices is 78.57%, risk of persons is 72.72% and risk of facility devices is 77.78%. As the risk recognition is based on the recognition of activity, especially most risk about a person is based on multiple activity and person recognition, the result may be considered acceptable. According to risk situation and risk degree, the accuracy of decision making for service provision is 100%. The approach is effective in risk detection and decision support for service in daily life.

#### 6.2.2. Time Response

**Figure 26:**
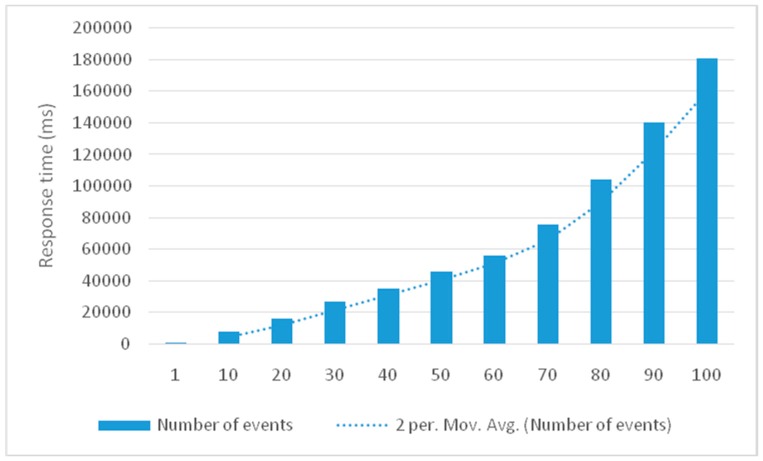
Response time of the approach. (per. Mov. Av: per Moving Average).

To test the time response of the approach, we calculated the time when the data were fed to the received command when various events occurred. The average response time to deal with a single event is 673 milliseconds. When dealing with several events occurring simultaneously, the response time increases with the number of events. Figure 26 indicates that the time taken linearly increases with the events to be processed during the first period. However, this variable increases rapidly after increasing the number of events. The results show that the total execution time is not too long and sufficient for safety decision support when the number of processed events is not high, which is suitable for a small neighbourhood. However, when many events require processing, such as that in a large neighbourhood, processing time will increase and the system should be further optimised. This problem will be the next addressed in our future research.

#### 6.2.3. Extensibility Evaluation

To evaluate if this approach is effective in pervasive service environments, the extendibility of the ontology proposed by us is tested.

If users want to add a new SH to the management system, the specific steps are:

(1) Add new home entity individual to “Home_entity” class. Its subclasses such as rooms in it are defined also;
(2) Add new home context individuals to the subclasses of “Person”, “Device”, “Sensor”, “Actuator” etc. according to the situation of new home;
(3) Add new manager individuals to the subclass of “Manager”;
(4) Link the property contact between new individuals, such as the linkage between “Device” and “Sensor”, “Person” with “Activity” and so on. Especially, the relationship between the monitored object with “Risk_Object”, “Risk_Event” and “Safe_Object” is very important;
(5) Related reasoning rules about the new home are selected.

After this, we tested the ontology and it runs very well. No single line of the previous code needs to be changed. If the approach is applied to other types of scenarios, such as hotels or apartment buildings, the process is similar, with only some small differences in management hierarchy.

In summary, the adding of new SHs to the system is very straightforward. The monitoring process can be directly applied to the new SHs. The approach has a good extendibility.

## 7. Conclusions

The ontology results are applied using a clear structure. The ontology adopts a natural assumption that is highly suitable for pervasive computing systems. The accurate expression of knowledge is essential when the risk is automatically monitored, identified and processed in SHs. SWRL is used to describe the reasoning rules for an OWL instance and to infer new knowledge.

This study proposes an approach based on ontology and SWRL rules for risk recognition and service decision support for SH management in a neighbourhood. We use the concept of ontology because it facilitates the integration and description of all entities which are presented in the SH environment. The structure and state information of SHs is encoded into the ontology, which can be used for the real-time monitoring of SHs. We found that the approach is suitable for decision support for the safety service provision of a small neighbourhood. We believe that the use of ontology allowed for a full description of the SH environment and made the system more open by allowing the addition or the removal of entities at any time according to the needs of management. Furthermore, the reasoning functions of decision support for safety service are improved though the combination with SWRL rules. A rule reasoning based on the context expression provided by the ontology can help us make intelligent decisions for safety service provision.

The prototype of the system is implemented using Protégé and Jess tools. We have evaluated the approach in terms of accuracy, time response and extendibility. Our case study shows that the proposed method effectively recognises the risk in daily life, and provides proper service according to the degree of risk. This approach is suitable for a pervasive environment and can be adopted in different SH styles.

In the future, we will improve the monitoring functions of the system to provide daily living assistance and health care. The accuracy of event recognition and the response time of the approach will be enhanced. In addition, the issue of privacy should be taken into account. The residents living in SHs should have the right to opt-out of the use of some sensor inputs.

## Figures and Tables

**Table 1:** Some property relationships between relevant classes from a home context perspective.

Property	Domain	Range	Functional Property
Locate_in	Person/Device/Sensor	Room	Functional
Monitored_by	Device/Person	Sensor	Inverse Functional
Sensing	Sensor	Device	Inverse Functional
has_monitored_state	Sensor	Status	Functional
operated_by	Electronic_device	Person	Functional
control	Actuator	Electronic_device	Functional
has_nature	Person	Person_nature	Functional
do_activity	Person	Activity	Functional

**Table 2:** Some properties’ relationships between relevant classes from a risk context perspective.

Property	Domain	Range	Functional Property
has_Risk	Home_unit	Risk	Functional
has_Risk_Degree	Home_unit	Risk_Degree	Functional
Sensing	Sensor	Device	Functional
relate_with	Device/Person	Risk_object	Functional
has_Time_interval	Risk_Degree	Time_interval	Functional

**Table 3:** Some property relationships between relevant classes from the perspective of service.

Property	Domain	Range	Functional Property
Providing_Service	Manager	Manage_service	Functional
managing_Area	Manager	Floor_building	Functional
Manual_service_provided_to	Manual_service	Home_unit	Functional
auto_service_provided_to	Auto_service	Actuator	Functional
message_service_provided_to	Message_service	Family_member	Functional
managing_Area	Manager	Floor_building	Functional
has_unit	Floor_building	Home_unit	Functional
Has_Rooms	Home_unit	Room	Functional
Person	Is_a	Family_member	Functional

**Table 4:** An example of class hierarchy related with a “cooking” activity.

Domain Class	Property	Range Class
Gas_oven	locate_in	Room(Kitchen)
Person; Electronic_device	monitored_by	Position_sensor; Device_sensor
Gas_oven	related_with	Cooking
Kitchen	has_sensor	Position_sensor
Kitchen ADL	locate_in	Kitchen
Position_sensor; Device_sensor	has_monitored_state	Person_status; Device_status
Person_status; Device_status	at_time	Time
Person_status; Device_status	has_Timeinterval	Time_interval

**Table 5:** Risk degree classification of different situations.

Degree	Description	Service	Service Provider
I	Slight: For example, some dangerous appliances are left operating for some time.	Notice message	Remote centre server
II	Light: For example, a baby crawls onto the balcony without the supervision of adults.	Warning message	Remote centre server
III	Moderate: For example, a dangerous appliance is still operating when the resident leaves his/her home.	Auto service Notice message	Actuator in SH with the control of the remote centre server
IV	Serious: For example, the resident has left home for a long time but forgot to lock the door.	Manual service Notice message	Managers of the neighbourhood

**Table 6:** Typical simple cases related to home safety.

Situation	Type	Service
Air conditioner is operating without anyone at home for a short time	Risk situations about electronic device	Notice message
Air conditioner is operating without anyone at home for a medium time		Warning service
TV is operating without anyone at home for a short time		Notice message
TV is operating without anyone at home for a long time		Auto service
Gas oven is operating without anyone in the kitchen for a medium time		Warning message
Gas oven is operating without anyone at home for a long time		Auto service
…		…
A baby goes to the balcony for a medium time	Risk situations about person	Warning message
A baby goes to the balcony for a long time		Manual service
A stranger is at home for a medium time		Warning message
A stranger is at home for a long time		Manual service
…		…
Tap is opened without anyone in the washing room for a medium time (resident is doing another activity)	Risk situations about facility device	warning service
Tap is opened without anyone at home for a long time		Manual service
Nobody is at home but the door is opened for a medium time		Warning message
Nobody is at home for a long time but the door is opened for a long time		Manual service
…		…

**Table 7:** Typical simple cases related to home safety.

**Risk Type**	**Number**
Risk situations about electronic device (ed)	28
Risk situations about person	22
Risk situations about facility device (fd)	18
**Service type**	**Number**
Notice message	36
Warning message	17
Auto service	9
Manual service	6
